# Cluster-Based Immunization Patterns in Diabetes Mellitus: Insights for Personalized Preventive Care

**DOI:** 10.3390/jpm15090441

**Published:** 2025-09-16

**Authors:** Teresa Gisinger, Alexandra Kautzky-Willer, Michael Leutner

**Affiliations:** Division of Endocrinology and Metabolism, Department of Internal Medicine III, Medical University of Vienna, Währinger Gürtel 18-20, 1090 Vienna, Austria; teresa.gisinger@meduniwien.ac.at (T.G.); alexandra.kautzky-willer@meduniwien.ac.at (A.K.-W.)

**Keywords:** diabetes mellitus, preventive care medicine, immunization

## Abstract

**Background**: We investigated immunization status and preventive care among diabetes mellitus (DM) patients by stratifying them into clinically distinct risk clusters based on comorbidities, reflecting a personalized medicine approach. **Methods**: Using the Austrian health interview survey 2019, we identified four groups: cluster 1 (DM, arterial hypertension (aHTN), dyslipidemia; *n* = 215), cluster 2 (DM, aHTN, dyslipidemia, obesity class II; *n* = 33), cluster 3 (DM, aHTN, dyslipidemia, depression; *n* = 65), and a control cohort (DM without hyperlipidemia, hypertension, depression, or obesity class II; *n* = 214). The cohorts were compared by chi^2^ tests. By logistic regression the association of the cluster-related variables and the vaccination status/preventive care variables were analyzed. **Results:** Significant differences in intact diphtheria immunization between the cohorts exist (cluster 1: 45.6%, cluster 2: 27.3%, cluster 3: 52.3%, control: 51.9%, *p*-value 0.047). Differences in intact tetanus (42.4% vs. 64%, *p* = 0.027) and diphtheria (27.3% vs. 51.9%, *p* = 0.013) immunization between cluster 2 and control cohort were investigated. Cluster 2 was negatively associated with tetanus (OR 0.83, *p* = 0.009) and diphtheria (OR 0.85, *p* = 0.018) immunization. Cluster 1 reports higher rates of fecal occult blood test (50.7% vs. 39.3%, *p* = 0.022) and cluster 2 reports a higher rate of colonoscopy (24.2% vs. 8.9%, *p* = 0.015) in comparison to the control cohort. **Conclusions**: A personalized medicine approach reveals that DM patients with specific comorbidity patterns, particularly those with hypertension, dyslipidemia, and obesity class II, have lower immunization rates—highlighting the need for targeted preventive strategies.

## 1. Introduction

Diabetes mellitus (DM) is a serious worldwide burden with enormous economic costs [1]. In 2024, the global costs on diabetes-related healthcare exceeded over USD 1 trillion [1]. Reports show that as of 2024, 589 million adults worldwide had diabetes mellitus, and this number is expected to reach 783 million by 2045 [1]. According to the Global Burden of Disease Study in 2013, diabetes mellitus ranked as the ninth major cause of reduced life expectancy [2]. For example, in 2021 diabetes mellitus led to 1.6 million deaths and 47% of all deaths occurred before the age of 70 years [3]. Even though the mortality rate of diabetes mellitus individuals is decreasing in the last decade, this cohort still reports higher risk compared to individuals without diabetes mellitus [4]. High rates of morbidity and mortality [2,5] in diabetes mellitus are largely attributable to its microvascular and macrovascular complications [6,7]. As known the risk of diabetes mellitus complications is increasing with the duration of the disease and worse glucose profiles [7]. Moreover, individuals with diabetes mellitus who suffer from comorbidities as dyslipidemia and hypertension had a higher frequency of diabetes complications [8]. Previous research has shown that diabetes mellitus influences cancer incidence, highlighting the critical need for preventive care within this population [9]. Furthermore studies report that hyperglycemia in patients with cancer leads to higher rates of adverse outcomes and events [10]. Moreover, diabetes mellitus can affect the immune system and thereby increases the risk of infections [11]. For example, an Austrian study demonstrated higher rates for pneumonia in individuals with diabetes mellitus [11]. The reason behind the alterations of the immune system and the immune response in individuals with diabetes mellitus is the high blood glucose levels [12]. Further it is known that in healthy subjects hyperglycemia is able to reduce neutrophil degranulation and interleukin-1α production, leading to increased inflammation-induced neutrophil activation and aggravated coagulation [13,14]. Moreover, in DM individuals polymorphonuclear leukocytes produce higher levels of reactive oxygen species, which might modulate natural killer cell function and therefore influence the risk of infections and malignancies [15,16]. Current findings concerning vaccination rates in DM individuals vary with good COVID-19 vaccination rate of 87.7% in a Chinese population, whereas a metanalysis found a high vaccination hesitancy of 27.8% in DM individuals in 2023 [17,18].

Because of the increased risk of complications in individuals with DM and comorbidities, it is essential to investigate and improve preventive care strategies tailored to individual patient profiles. Recent work has applied advanced analytic methods such as clustering and machine learning to better characterize the heterogeneity of diabetes mellitus and its comorbidities. For example, deep embedding and clustering approaches in large cohorts have revealed distinct patient subgroups with differing disease trajectories and complication risks [19,20,21]. A personalized medicine approach recognizes that preventive and immunization needs may differ significantly depending on the presence and type of comorbid conditions. Hence, the aim of the present study was to investigate the status of preventive care and immunization in individuals with DM without comorbidities, as well as in cohorts with different comorbidities, to support more targeted and individualized preventive healthcare strategies.

## 2. Materials and Methods

In this study the data used from The Austrian Health Information Survey (AT-HIS) were obtained after a written application from Statistik Austria. AT-HIS was part of the larger European Health Information Survey (EHIS) [22]. This survey was conducted in 2019 with Computer Assisted Telephone Interviewing and surveys in written form after obtaining informed consent from all the participants. Informed Consent for our study is not required for the use of data from The Austrian Health Information Survey (AT-HIS) with anonymous information. For our analysis we only used individuals diagnosed with diabetes mellitus (*n* = 964, 52.2% males, 47.8% females). Further we defined disease clusters that are already described to have a higher diabetes mellitus complication rate [8]. Cluster 1 included individuals with diabetes mellitus, hypertension, and dyslipidemia. Cluster 2 consists of individuals with diabetes mellitus, hypertension, dyslipidemia, and obesity class II (defined as a BMI of more than or equal to 35 kg/m^2^). Cluster 3 consists of individuals with diabetes mellitus, hypertension, dyslipidemia, and depression. Our control cohort (DM) consisted of patients with diabetes mellitus without hypertension, dyslipidemia, depression, or BMI more than or equal to 35 kg/m^2^.

Outcome measures included variables related to the vaccination status and to preventive medical examination. In the AT-HIS survey the question and the available answers were as follows “Last time of blood pressure measurement by a health care professional?”, “Last time of blood cholesterol measurements of a health care professional?”, “Last time of fecal occult blood test?”, “Last time of colonoscopy?”, “Last time of mammography (breast X-ray)?”, “Last time of cervical smear?”, “Last flu vaccination”—Available answer “Within the past 12 months”, “over 12 months ago”. Further questions concerning intact immunization status (tetanus, diphtheria, Polio, FSME, pneumococcus) were dichotomous.

### Statistical Analysis

Descriptive results were reported as frequency and percentage for categorical variables. The different cohorts were compared using a chi^2^ test. First a comparison over all cohorts was carried out. Afterwards each cohort was compared to all other cohorts individually. If the observation number was under five a Fisher exact test was performed. The relationship between the disease clusters and the preventive care variables and vaccination status were assessed using a logistic regression model for each study group. In order to test the model fit a Hosmer–Lemeshow was performed (Supplementary Table S4). Data analysis was performed using R software (Version 4.0.3). *p*-values < 0.05 were considered to be statistically significant.

## 3. Results

### 3.1. Comparison of All Risk Clusters

In Table 1 the descriptive analyses of the control cohort and the risk clusters are reported. Comparing all study cohorts at the same time, only differences in sex, age, and diphtheria immunization can be seen.

### 3.2. Cluster 2 Reports Worse Immunization Status—Individual Comparison of Each Cluster

Moreover, each cohort was compared to all the other cohorts individually (see Supplementary Materials Tables S1–S3). Compared to the control cohort, cluster 2 showed lower immunization rates for tetanus and diphtheria but a higher frequency of colonoscopy. When compared with cluster 1, cluster 2 again demonstrated lower tetanus immunization rates and more frequent colonoscopy. In comparison with cluster 3, cluster 2 exhibited lower diphtheria immunization. Additionally, cluster 1 underwent fecal occult blood testing more often than the control cohort. No relevant differences were observed between cluster 3 and the control cohort, nor between clusters 1 and 3.

### 3.3. Association of Preventive Care/Immunization Status and Risk Clusters

**Figure 1:**
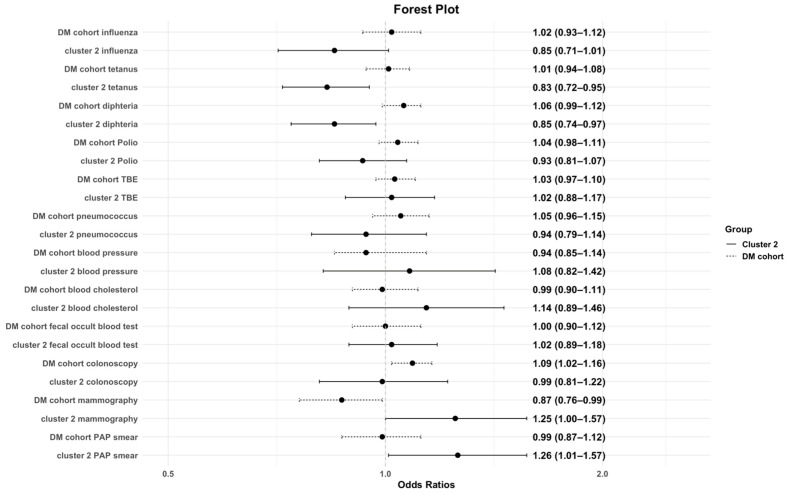
Association of preventive care/immunization status and the DM cohort and cluster 2. This figure illustrates the odds ratios with corresponding 95% confidence intervals for the likelihood of being immunized and undertaking preventive care examination among cluster 2 and the control group. An OR below 1 indicates lower odds of immunization, whereas an OR above 1 indicates higher odds. The dashed line represents the control cohort.

Further the association of immunization status and preventive care on the characteristics of the study cohorts were investigated (see Table 2 and Figure 1). Cluster 2 was negatively associated with tetanus and diphtheria immunization but positively associated with colonoscopy and PAP smear. In the control cohort, colonoscopy was more frequent, whereas mammography was less frequent. Cluster 1 showed positive associations with fecal occult blood testing and colonoscopy. Cluster 3 was positively associated with fecal occult blood testing.

## 4. Discussion

In summary the worst intact immunization was investigated in cluster 2, which were individuals with diabetes mellitus, hypertension, dyslipidemia, and obesity grade 2. Compared to the control group, individuals with diabetes mellitus, hypertension, dyslipidemia, and grade 2 obesity were less likely to have complete tetanus and diphtheria immunization. Nevertheless, the rate of preventive care usage as fecal occult blood test or colonoscopy was higher in the risk clusters compared to the control cohort. When we examined individual comorbidities, some associations were observed. The cluster differences highlight meaningful patterns and might suggest that comorbidity clusters can help identify patient subgroups with different preventive care and immunization needs, supporting a personalized medicine approach.

The rate of complicated courses of infectious diseases are claimed to be higher in diabetes mellitus patients compared to individuals without diabetes mellitus [23]. The increased susceptibility and prevalence rates of infections in diabetes mellitus individuals might be caused by a disturbed innate immune response [23]. Higher infection risk of various viruses including Kaposi’s sarcoma-associated herpesvirus, severe acute respiratory syndrome coronavirus, Middle East respiratory syndrome coronavirus, hepatitis C virus, and West Nile encephalitis is reported in individuals with diabetes mellitus [24,25,26,27]. Furthermore, the previous literature analyzed that individuals with diabetes mellitus are at increased risk of influenza- and pneumonia-mediated morbidity and mortality [11,28]. In our study, influenza immunization coverage ranged between 14% and 18% across all cohorts, which is notably lower than reported in other settings—for example, a study in Saudi Arabia found that 43.5% of individuals with diabetes mellitus had received the influenza vaccine [29]. Also, in a Korean study population almost 60% of diabetes mellitus patients were vaccinated against influenza [30]. The Austrian Health Interview Survey, as well as the Hungarian Health Information Survey, were part of the European Health Information Survey with a similar protocol. The Hungarian study concluded that even though the data are self-reported, the influenza vaccination data could be considered valid due to the strict regulations applied during data collection [31]. The past literature already found a high vaccine hesitance in Austria caused by the fear of adverse events, doubt of effectiveness, and distrust towards the pharmaceutical industry [32]. Furthermore, our results report lower intact immunization (tetanus: 42.4% versus 64.0%, diphtheria: 27.3% versus 51.9%) in an even more vulnerable diabetes mellitus risk cluster including individuals with diabetes mellitus, hypertension, dyslipidemia, and obesity grade 2. This finding could be related to the possibility that individuals with obesity may be less likely to attend medical appointments if they perceive or experience negative attitudes or stereotypes from healthcare providers, which can contribute to stress, mistrust, and lower adherence to care [33]. It is postulated that healthcare providers might view obesity as an avoidable risk factor that hinders their ability to treat and prevent disease and therefore they have a negative attitude towards them [33]. This negative attitude further reduces the likelihood that individuals with obesity attend medical check-ups [33]. Even though previous studies report that diabetes mellitus individuals with comorbidities have a higher influenza immunization rate [34,35]. Still no data concerning tetanus and diphtheria immunization in the current literature can be found. Concerning obesity, it is already known that obesity is related to a higher frequency of community-acquired infections and is accompanied by longer hospitalization rate [36,37]. Obesity may alter the immune system as excess adipose tissue is associated with a chronic inflammation state and leads to cell necrosis and dysfunction [36]. Further visceral adipose tissue produces more cytokines including tumor necrosis factor α and interleukin 6 and 1β, which weaken the immune response against infectious diseases [36]. During the COVID-19 pandemic, it has been investigated that higher infection rates and worse disease outcomes were reported in diabetes mellitus and obese patients [38]. Furthermore, the COVID-19 mortality was 3 times higher in type 2 diabetes mellitus individuals with a BMI of 30 kg/m^2^ compared to other individuals at the ICU department with COVID-19 [38]. Also, dyslipidemia might affect the immune system by modulating T regulatory (Treg) cell metabolism. Within Treg cells, mTORC1 supports metabolic regulation by enhancing glycolysis in T cells [39]. Nevertheless, cholesterol accumulation might inhibit mTORC1 [40]. In addition, dyslipidemia influences Treg cell metabolism through the activation of peroxisome proliferator-activated receptors, lipid-sensitive transcription factors that regulate cellular metabolism [41]. Also, in hypertension, the influence of the immune system gained importance. It is postulated that numerous pro-inflammatory markers are elevated in hypertension such as interleukin-17, interleukin-6, interleukin-1β, tumor necrosis factor-α, and inteurleukin-23 [42,43]. Therefore, intact immunization is crucial in obese type 2 diabetes mellitus individuals with hypertension and dyslipidemia.

Previous work could already report that the defined risk clusters have worse diabetes mellitus outcome [8]. More specifically the combination of diabetes mellitus, hypertension, and dyslipidemia had higher frequencies of macrovascular and microvascular complications [8]. In the previously described disease clusters higher rates of cardiovascular disease, atherosclerosis, and amputation of legs compared to the diabetes mellitus cohort can be found [8]. Moreover, previous work investigated that individuals with diabetes mellitus and major depressive disorder are associated with worse glycemic control than the general diabetes mellitus cohort without depression [44]. That is why sufficient preventive medical exanimations and check-ups are of importance especially in individuals with diabetes mellitus and other comorbidities. However, the current literature suggests that individuals with depression are less likely to obtain influenza vaccination and preventive care [45]. Individuals with depressive symptoms may be less likely to engage in preventive healthcare due to low motivation and negative attitudes, including concerns about the safety and efficacy of vaccines [46]. In all our study cohorts as well as the control cohort we can report cholesterol measurements rates of over 90%, which is similar to findings in the US with 83% of type 2 diabetes mellitus patients obtaining cholesterol measurements [47]. In our study higher frequency of fecal occult blood test in individuals with diabetes mellitus, hypertension, and hyperlipidemia was found compared to the control cohort. Moreover, a higher frequency of colonoscopy in individuals with diabetes mellitus, hypertension, hyperlipidemia, and obesity grade 2 was investigated. These findings might be explained due to a higher frequency of doctor visits or hospitalization in individuals with diabetes mellitus and comorbidities compared to individuals with diabetes mellitus without comorbidities. Nevertheless, the previous literature reported that diabetes mellitus individuals with an age over 50 years have a higher rate of doctors’ visits but still undergo less preventive care examinations as mammographies compared to their non-diabetes mellitus counterparts [48]. Furthermore, it is known that individuals with obesity have a hesitancy to vaccination even though they are at higher risk [49], which might explain the lower vaccination rate observed in cluster 2. Nevertheless, other studies suggest higher vaccination rate in obesity caused by the awareness of higher virus-related complications and higher rate of doctors’ visits. Conversely, other studies have reported higher vaccination uptake among people with obesity, potentially driven by greater awareness of infection-related risks and more frequent healthcare interactions [50].

This study has some limitations. First, it relies on self-reported data, which may introduce recall bias or result in over- or underestimation of disease prevalence, healthcare utilization, and immunization rates. Second, the data do not distinguish between type 1 and type 2 diabetes mellitus. Although it is reasonable to assume that most cases in our cohort represent type 2 diabetes, given its substantially higher global prevalence [51,52], we recognize that comorbidity patterns, healthcare utilization, and preventive care needs can differ between type 1 and type 2 diabetes. Therefore, the lack of differentiation may have influenced our findings, and the results should be interpreted with this limitation in mind. Thirdly, some of our reported odds ratios are close to 1, and thus their clinical relevance at the individual level may be limited. Still, even modest effect sizes can be meaningful in public health contexts when applied across large populations, or when combined with other risk factors. Fourthly, the cross-sectional design of this study inhibit any causal inferences, and the small sample size in cluster 2 may result in unstable estimates and reduced precision and robustness. Finally, because the data were obtained from Austria, unmeasured confounders—such as socioeconomic status, healthcare access, and regional differences—may have influenced the observed associations and might limit generalizability outside Austria.

Previous studies have indicated that certain groups, such as the elderly, adolescents, and individuals with lower socioeconomic status, are less likely to respond to health surveys in Austria. This non-response can lead to underrepresentation of these populations, potentially skewing the results [53].

## 5. Conclusions

The novel strength of this study lies in its focus on a diabetes mellitus cohort without comorbidities, compared to various diabetes mellitus cohorts with comorbidities, across a range of immunizations and preventive medical examinations. While numerous studies have examined influenza immunization, feet and eye examinations and primary care usage of diabetes mellitus individuals, few have systematically compared various immunization rates and preventive care measures simultaneously across subgroups defined by comorbidity status. By highlighting these differences, our study provides valuable insights that can inform more nuanced and individualized care strategies. Ultimately, this research supports the development of a more personalized treatment approach that better addresses the specific preventive care needs of both the general and more vulnerable DM populations.

## Figures and Tables

**Table 1:** Baseline characteristics and comparison of cohorts.

Variable	DM (*n* = 214)	Cluster 1 (*n* = 215)	Cluster 2 (*n* = 33)	Cluster 3 (*n* = 65)	*p*-Value Chi^2^ Test
Age:					<0.001
- 15–49	28 13.4%	2 1% *	2 6%	5 7.9%	
- 50–74	126 59.0%	119 55.4% *	22 67%	31 47.4%	
- >74	59 27.5%	93 43.2% *	9 27%	29 44.7%	
Sex:					<0.001
- male	130 60.7%	111 51.6%	16 48.5% ^#^	21 32.3% *	
- female	84 39.3%	104 48.4%	17 51.5% ^#^	44 67.7% *	
Last influenza shot in the past 12 months	30 14.0%	38 17.7%	6 18.2%	9 13.8%	0.704
Intact tetanus immunization	137 64.0%	134 62.3% ^+^	14 42.4% *	40 61.5%	0.112
Intact diphtheria immunization	111 51.9%	98 45.6%	9 27.3% *^,#^	34 52.3%	0.047
Intact Polio immunization	99 46.3%	94 43.7%	11 33.3%	28 43.1%	0.545
Intact TBE immunization	126 58.9%	116 54.0%	13 39.4%	30 46.2%	0.081
Intact pneumococcus immunization	27 12.6%	32 14.9%	3 9.1%	7 10.8%	0.781
Last blood pressure measurement in the past 12 months	192 89.7%	203 94.4%	32 97.0%	61 93.8%	0.245
Last blood cholesterol measurement in the past 12 months	194 90.7%	203 94.4%	32 97.0%	61 93.8%	0.413
Last fecal occult blood test in the past 12 months	84 39.3%	109 50.7% *	15 45.5%	32 49.2%	0.108
Last colonoscopy in the past 12 months	19 8.9%	25 11.6% ^+^	8 24.2% *	5 7.7%	0.072
Last mammogram in the past 12 months	22 26.2%	28 26.9%	5 29.4%	13 29.5%	0.953
Last PAP smear in the past 12 months	35 41.7%	30 14.7%	4 23.5%	15 34.1%	0.234

Frequency and percentage of immunization status and preventive medical examination in the DM cohort, clusters 1, 2, and 3. Further, a chi^2^ test was performed in order to compare all 4 study cohorts and the *p*-values are reported. Moreover, each cohort was compared with each other cohort by a chi^2^ test. * indicates a significant difference between the DM cohort and the specific cluster. ^+^ indicates a significant difference between the clusters 1 and 2. ^#^ indicates a significant difference between the clusters 2 and 3.

**Table 2:** Association of study cohorts and immunization status/preventive care.

Variable	DM	Cluster 1	Cluster 2	Cluster 3
Last influenza shot in the past 12 months	1.02 [0.93–1.12] (0.688)	1.12 [1.00–1.26] (0.059)	0.85 [0.71–1.01] (0.064)	1.39 [0.97–1.97] (0.077)
Intact tetanus immunization	1.01 [0.94–1.08] (0.836)	1.04 [0.94–1.14] (0.465)	0.83 [0.72–0.95] (0.009)	1.02 [0.79–1.30] (0.901)
Intact diphtheria immunization	1.06 [0.99–1.12] (0.077)	1.05 [0.95–1.14] (0.343)	0.85 [0.74–0.97] (0.018)	1.12 [0.88–1.44] (0.363)
Intact Polio immunization	1.04 [0.98–1.11] (0.183)	1.03 [0.94–1.13] (0.529)	0.93 [0.81–1.07] (0.293)	1.05 [0.82–1.36] (0.685)
Intact TBE immunization	1.03 [0.97–1.10] (0.324)	0.98 [0.90–1.08] (0.739)	1.02 [0.88–1.17] (0.830)	0.90 [0.71–1.15] (0.417)
Intact pneumococcus immunization	1.05 [0.96–1.15] (0.270)	1.09 [0.97–1.23] (0.148)	0.94 [0.79–1.14] (0.547)	1.33 [0.89–1.99] (0.173)
Last blood pressure measurement in the past 12 months	0.94 [0.85–1.05] (0.278)	1.15 [0.95–1.39] (0.166)	1.08 [0.82–1.42] (0.600)	1.14 [0.75–1.73] (0.551)
Last blood cholesterol measurement in the past 12 months	0.99 [0.90–1.10] (0.947)	1.18 [0.96–1.45] (0.114)	1.14 [0.89–1.46] (0.308)	1.06 [0.72–1.56] (0.777)
Last fecal occult blood test in the past 12 months	1.00 [0.90–1.12] (0.963)	1.52 [1.15–2.01] (0.004)	1.02 [0.72–1.35] (0.729)	1.34 [1.05–1.71] (0.021)
Last colonoscopy in the past 12 months	1.09 [1.02–1.16] (0.012)	1.14 [1.05–1.25] (0.003)	0.99 [0.81–1.22] (0.937)	0.78 [0.53–1.15] (0.218)
Last mammogram in the past 12 months	0.87 [0.76–0.99] (0.049)	1.07 [0.95–1.20] (0.258)	1.25 [1.00–1.57] (0.053)	1.20 [0.87–1.65] (0.276)
Last PAP smear in the past 12 months	0.99 [0.87–1.12] (0.852)	1.07 [0.95–1.20] (0.258)	1.26 [1.01–1.57] (0.042)	1.22 [0.92–1.63] (0.185)

The relationship between the DM and risk clusters and the preventive care variables, vaccination status, satisfaction with healthcare services, and unmet healthcare needs were assessed using a logistic regression model. The odds ratios for the logistic regression are reported. Furthermore, the 95% confidence interval are reported in the squared brackets and the *p*-values in the round brackets.

## Data Availability

Data can be obtained after an application at Statistik Austria.

## Supplementary Materials

The following supporting information can be downloaded at: https://www.mdpi.com/article/10.3390/jpm15090441/s1, Figure S1: Flow diagram of study participants; Table S1: Comparison of DM cohort vs. risk cohorts, Table S2: Comparison of cluster 1 vs. risk cohorts, Table S3: Comparison of cluster 2 vs. risk cohorts, Table S4: Model Fit Assessment.

## References

[1] IDF (2025). IDF Diabetes Atlas 11th Edition. https://diabetesatlas.org/resources/idf-diabetes-atlas-2025/#:~:text=The%20IDF%20Diabetes%20Atlas%2011th,territory%2C%20for%202024%20and%202050.

[2] The Lancet (2015). Global, regional, and national age-sex specific all-cause and cause-specific mortality for 240 causes of death, 1990–2013: A systematic analysis for the Global Burden of Disease Study 2013. Lancet.

[3] World Health Organization (2024). Diabetes. https://www.who.int/news-room/fact-sheets/detail/diabetes#:~:text=In%202021%2C%20diabetes%20was%20the,of%20cardiovascular%20deaths%20(1).

[4] Gregg E.W., Zhuo X., Cheng Y.J., Albright A.L., Narayan K.M., Thompson T.J. (2014). Trends in lifetime risk and years of life lost due to diabetes in the USA, 1985–2011: A modelling study. Lancet Diabetes Endocrinol..

[5] Federation I.D. IDF Diabetes Atlas. https://diabetesatlas.org.

[6] Litwak L., Goh S.Y., Hussein Z., Malek R., Prusty V., Khamseh M.E. (2013). Prevalence of diabetes complications in people with type 2 diabetes mellitus and its association with baseline characteristics in the multinational A1chieve study. Diabetol. Metab. Syndr..

[7] Gregg E.W., Sattar N., Ali M.K. (2016). The changing face of diabetes complications. Lancet Diabetes Endocrinol..

[8] Leutner M., Haug N., Bellach L., Dervic E., Kautzky A., Klimek P., Kautzky-Willer A. (2021). Risk of Typical Diabetes-Associated Complications in Different Clusters of Diabetic Patients: Analysis of Nine Risk Factors. J. Pers. Med..

[9] Coughlin S.S., Calle E.E., Teras L.R., Petrelli J., Thun M.J. (2004). Diabetes mellitus as a predictor of cancer mortality in a large cohort of US adults. Am. J. Epidemiol..

[10] Hammer M., Storey S., Hershey D.S., Brady V.J., Davis E., Mandolfo N., Bryant A.L., Olausson J. (2019). Hyperglycemia and Cancer: A State-of-the-Science Review. Oncol. Nurs. Forum.

[11] Leutner M., Kaleta M., Bellach L., Kautzky A., Thurner S., Klimek P., Kautzky-Willer A. (2021). Insulin as Monotherapy and in Combination with Other Glucose-Lowering Drugs Is Related to Increased Risk of Diagnosis of Pneumonia: A Longitudinal Assessment over Two Years. J. Pers. Med..

[12] Ferlita S., Yegiazaryan A., Noori N., Lal G., Nguyen T., To K., Venketaraman V. (2019). Type 2 Diabetes Mellitus and Altered Immune System Leading to Susceptibility to Pathogens, Especially Mycobacterium tuberculosis. J. Clin. Med..

[13] Stegenga M.E., van der Crabben S.N., Blümer R.M., Levi M., Meijers J.C., Serlie M.J., Tanck M.W., Sauerwein H.P., van der Poll T. (2008). Hyperglycemia enhances coagulation and reduces neutrophil degranulation, whereas hyperinsulinemia inhibits fibrinolysis during human endotoxemia. Blood.

[14] Stegenga M.E., van der Crabben S.N., Dessing M.C., Pater J.M., van den Pangaart P.S., de Vos A.F., Tanck M.W., Roos D., Sauerwein H.P., van der Poll T. (2008). Effect of acute hyperglycaemia and/or hyperinsulinaemia on proinflammatory gene expression, cytokine production and neutrophil function in humans. Diabet. Med..

[15] Hand W.L., Hand D.L., Vasquez Y. (2007). Increased polymorphonuclear leukocyte respiratory burst function in type 2 diabetes. Diabetes Res. Clin. Pract..

[16] Moretta A., Bottino C., Mingari M.C., Biassoni R., Moretta L. (2002). What is a natural killer cell?. Nat. Immunol..

[17] Li H., Ping F., Li X., Wang Z., Xiao J., Jiang H., Xue Y., Quan J., Yao H., Zheng X. (2023). COVID-19 vaccine coverage, safety, and perceptions among patients with diabetes mellitus in China: A cross-sectional study. Front. Endocrinol..

[18] Bianchi F.P., Stefanizzi P., Martinelli A., Brescia N., Tafuri S. (2023). COVID-19 vaccination hesitancy in people affected by diabetes and strategies to increase vaccine compliance: A systematic narrative review and meta-analysis. Vaccine.

[19] Munoz-Farre A., Poulakakis-Daktylidis A., Kothalawala D.M., Rodriguez-Martinez A. (2023). Interpreting deep embeddings for disease progression clustering. arXiv.

[20] Schaar C.L.a.M.v.d. (2020). Temporal Phenotyping using Deep Predictive Clustering of Disease Progression. arXiv.

[21] Mottalib M.M., Jones-Smith J.C., Sheridan B., Beheshti R. (2023). Subtyping patients with chronic disease using longitudinal BMI patterns. IEEE J. Biomed. Health Inform..

[22] Austria S. (2020). Die Österreichische Gesundheitsbefragung. https://www.statistik.at/services/tools/services/publikationen/detail/848.

[23] Daryabor G., Atashzar M.R., Kabelitz D., Meri S., Kalantar K. (2020). The Effects of Type 2 Diabetes Mellitus on Organ Metabolism and the Immune System. Front. Immunol..

[24] Alraddadi B.M., Watson J.T., Almarashi A., Abedi G.R., Turkistani A., Sadran M., Housa A., Almazroa M.A., Alraihan N., Banjar A. (2016). Risk Factors for Primary Middle East Respiratory Syndrome Coronavirus Illness in Humans, Saudi Arabia, 2014. Emerg. Infect. Dis..

[25] Yang J.K., Feng Y., Yuan M.Y., Yuan S.Y., Fu H.J., Wu B.Y., Sun G.Z., Yang G.R., Zhang X.L., Wang L. (2006). Plasma glucose levels and diabetes are independent predictors for mortality and morbidity in patients with SARS. Diabet. Med..

[26] Villar L.M., Geloneze B., Vasques A.C.J., Pires M.L.E., Miguel J.C., da Silva E.F., Marques V.A., Scalioni L.P., Lampe E. (2019). Prevalence of hepatitis B and hepatitis C among diabetes mellitus type 2 individuals. PLoS ONE.

[27] Kumar M., Roe K., Nerurkar P.V., Namekar M., Orillo B., Verma S., Nerurkar V.R. (2012). Impaired virus clearance, compromised immune response and increased mortality in type 2 diabetic mice infected with West Nile virus. PLoS ONE.

[28] Li S., Wang J., Zhang B., Li X., Liu Y. (2019). Diabetes Mellitus and Cause-Specific Mortality: A Population-Based Study. Diabetes Metab. J..

[29] Alsufyani S.A. (2022). Acceptance Rate of Influenza Vaccination Among Patients with Type II Diabetes. J. Family Med. Prim. Care.

[30] Lee D.H., Yang B., Gu S., Kim E.G., Kim Y., Kang H.K., Choe Y.H., Jeon H.J., Park S., Lee H. (2023). Influenza vaccination trend and related factors among patients with diabetes in Korea: Analysis using a nationwide database. Front. Endocrinol..

[31] Szőllősi G.J., Pataki J., Virágh A., Bányai G., Boruzs K., Bíró K., Dombrádi V. (2024). Influenza Vaccination Coverage among People with Self-Reported Cardiovascular Diseases-Findings from the Hungarian Implementation of the European Health Interview Survey. Vaccines.

[32] Sandhofer M.J., Robak O., Frank H., Kulnig J. (2017). Vaccine hesitancy in Austria: A cross-sectional survey. Wien. Klin. Wochenschr..

[33] Phelan S.M., Burgess D.J., Yeazel M.W., Hellerstedt W.L., Griffin J.M., van Ryn M. (2015). Impact of weight bias and stigma on quality of care and outcomes for patients with obesity. Obes. Rev..

[34] Yu M.C., Chou Y.L., Lee P.L., Yang Y.C., Chen K.T. (2014). Influenza vaccination coverage and factors affecting adherence to influenza vaccination among patients with diabetes in Taiwan. Hum. Vaccin. Immunother..

[35] Jimenez-Trujillo I., López-de Andrés A., Hernández-Barrera V., Carrasco-Garrido P., Santos-Sancho J.M., Jiménez-García R. (2013). Influenza vaccination coverage rates among diabetes sufferers, predictors of adherence and time trends from 2003 to 2010 in Spain. Hum. Vaccin. Immunother..

[36] Pugliese G., Liccardi A., Graziadio C., Barrea L., Muscogiuri G., Colao A. (2022). Obesity and infectious diseases: Pathophysiology and epidemiology of a double pandemic condition. Int. J. Obes..

[37] Harpsøe M.C., Nielsen N.M., Friis-Møller N., Andersson M., Wohlfahrt J., Linneberg A., Nohr E.A., Jess T. (2016). Body Mass Index and Risk of Infections Among Women in the Danish National Birth Cohort. Am. J. Epidemiol..

[38] Pérez-Cruz E., Castañón-González J.A., Ortiz-Gutiérrez S., Garduño-López J., Luna-Camacho Y. (2021). Impact of obesity and diabetes mellitus in critically ill patients with SARS-CoV-2. Obes. Res. Clin. Pract..

[39] Shi L.Z., Wang R., Huang G., Vogel P., Neale G., Green D.R., Chi H. (2011). HIF1alpha-dependent glycolytic pathway orchestrates a metabolic checkpoint for the differentiation of TH17 and Treg cells. J. Exp. Med..

[40] Cheng H.Y., Gaddis D.E., Wu R., McSkimming C., Haynes L.D., Taylor A.M., McNamara C.A., Sorci-Thomas M., Hedrick C.C. (2016). Loss of ABCG1 influences regulatory T cell differentiation and atherosclerosis. J. Clin. Investig..

[41] Bensinger S.J., Tontonoz P. (2008). Integration of metabolism and inflammation by lipid-activated nuclear receptors. Nature.

[42] Cai T., Zhang Y., Ho Y.L., Link N., Sun J., Huang J., Cai T.A., Damrauer S., Ahuja Y., Honerlaw J. (2018). Association of Interleukin 6 Receptor Variant With Cardiovascular Disease Effects of Interleukin 6 Receptor Blocking Therapy: A Phenome-Wide Association Study. JAMA Cardiol..

[43] Carranza-Leon D.A., Oeser A., Wu Q., Stein C.M., Ormseth M.J., Chung C.P. (2020). Ambulatory blood pressure in patients with systemic lupus erythematosus: Association with markers of immune activation. Lupus.

[44] Petrak F., Baumeister H., Skinner T.C., Brown A., Holt R.I.G. (2015). Depression and diabetes: Treatment and health-care delivery. Lancet Diabetes Endocrinol..

[45] Baek S.U., Yoon J.H. (2024). Association between depressive symptoms and participation in influenza vaccination and health checkups: Findings from the Korea National Health and Nutrition Examination Survey. Gen. Hosp. Psychiatry.

[46] Nguyen K.H., Chen S., Morris K., Chui K., Allen J.D. (2022). Mental health symptoms and association with COVID-19 vaccination receipt and intention to vaccinate among adults, United States. Prev. Med..

[47] Wittman J.T., Bullard K.M., Benoit S.R. (2023). Trends in Preventive Care Services Among U.S. Adults With Diagnosed Diabetes, 2008-2020. Diabetes Care.

[48] Gisinger T., Kautzky-Willer A., Leutner M. (2022). Need for improving immunization status and preventive care in diabetes mellitus patients. Wien. Klin. Wochenschr..

[49] Kizilkaya M.C., Kilic S.S., Oncel D., Mamidanna S., Daliparty V., Yilmaz S., Bozkurt M.A., Sibic O., Sayan M. (2023). Barriers to Coronavirus Disease 19 vaccination in patients wit h obesity. Am. J. Surg..

[50] Harris J.A., Moniz M.H., Iott B., Power R., Griggs J.J. (2016). Obesity and the receipt of influenza and pneumococcal vaccination: A systematic review and meta-analysis. BMC Obes..

[51] Lascar N., Brown J., Pattison H., Barnett A.H., Bailey C.J., Bellary S. (2018). Type 2 diabetes in adolescents and young adults. Lancet Diabetes Endocrinol..

[52] Maahs D.M., West N.A., Lawrence J.M., Mayer-Davis E.J. (2010). Epidemiology of type 1 diabetes. Endocrinol. Metab. Clin. N. Am..

[53] Hintzpeter B., Finger J.D., Allen J., Kuhnert R., Seeling S., Thelen J., Lange C. (2019). European Health Interview Survey (EHIS) 2—Background and study methodology. J. Health Monit..

