# Supplementary material for: Cluster-Based Immunization Patterns in Diabetes Mellitus: Insights for Personalized Preventive Care
Source: J Pers Med. 2025 Sep 16;15(9):441. doi: 10.3390/jpm15090441 (PMC12470910; doi:10.3390/jpm15090441)
Supplement: Supplementary file 1 [file jpm-15-00441-s001.zip › jpm-3819615-supplementary.pdf]

Supplementary Materials

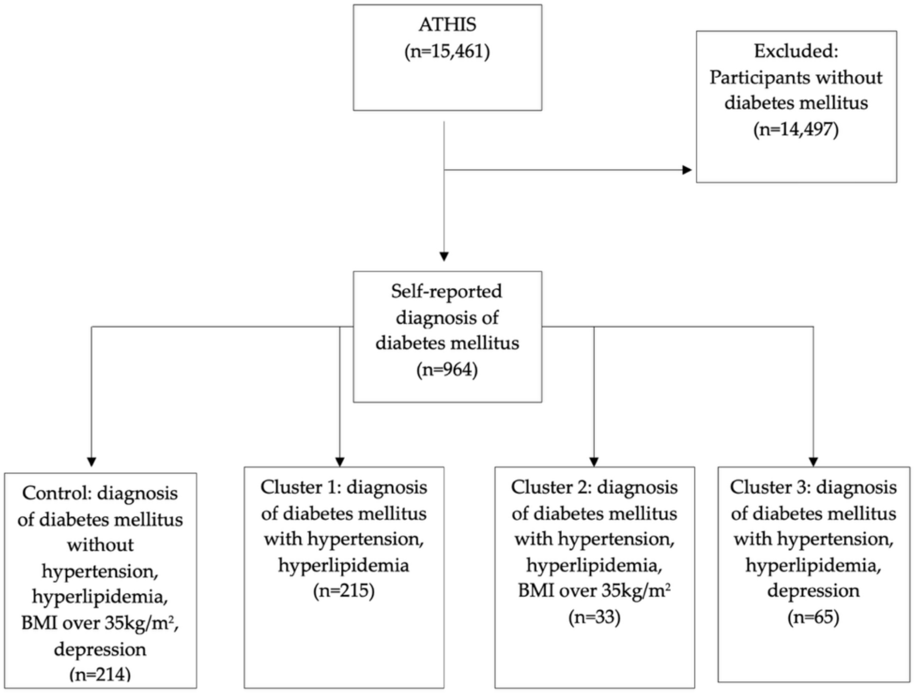

Supplementary Figure S1. Flow Diagram of Study Participants

This flow diagram reports study participant selection. ATHIS=Austrian Health Information Survey.

Supplementary Table S1. Comparison of DM cohort vs. risk cohorts

Variable	DM vs. Cluster 1	DM vs. Cluster 2	DM vs. Cluster 3
Last influenza shot in the past 12 months	0.366	0.595	1.000
Intact tetanus immunization	0.891	**0.027**	0.794
Intact diphtheria immunization	0.263	**0.013**	1.000
Intact Polio immunization	0.697	0.212	0.711
Intact TBE immunization	0.379	0.052	0.088
Intact pneumococcus immunization	0.942	0.583	0.639
Last blood pressure measurement in the past 12 months	0.105	0.331	0.448
Last blood cholesterol measurement in the past 12 months	0.194	0.326	0.582
Last blood sugar measurement in the past 12 months	**0.020**	0.483	1.000
Last fecal occult blood test in the past 12 months	**0.022**	0.627	0.199
Last colonoscopy in the past 12 months	0.436	**0.015**	0.963
Last mammogram in the past 12 months	1.000	0.770	0.845
Last PAP smear in the past 12 months	0.092	0.260	0.520

A chi^2 test was done in order to compare the DM cohort to the risk cohorts and the p-values are reported.

Supplementary Table S2. Comparison of cluster 1 vs. other risk cohorts

Variable	Cluster 1 vs. Cluster 2	Cluster 1 vs. Cluster 3
Last influenza shot in the past 12 months	1.000	0.593
Intact tetanus immunization	**0.038**	0.923
Intact diphtheria immunization	0.061	0.493
Intact Polio immunization	0.318	0.971
Intact TBE immunization	0.154	0.302
Intact pneumococcus immunization	0.583	0.532
Last blood pressure measurement in the past 12 months	0.330	0.770
Last blood cholesterol measurement in the past 12 months	0.326	0.770
Last blood sugar measurement in the past 12 months	0.483	0.135
Last fecal occult blood test in the past 12 months	0.709	0.948
Last colonoscopy in the past 12 months	**0.015**	0.503
Last mammogram in the past 12 months	0.770	0.901
Last PAP smear in the past 12 months	0.185	0.661

A chi^2 test was done in order to compare the DM cohort to the risk cohorts and the p-values are reported.

Supplementary Table S3. Comparison of cluster 2 vs. cluster 3

Variable	Cluster 2 vs. Cluster 3
Last influenza shot in the past 12 months	0.790
Intact tetanus immunization	0.113
Intact diphtheria immunization	**0.032**
Intact Polio immunization	0.476
Intact TBE immunization	0.673
Intact pneumococcus immunization	0.538
Last blood pressure measurement in the past 12 months	0.770
Last blood cholesterol measurement in the past 12 months	0.770
Last blood sugar measurement in the past 12 months	0.135
Last fecal occult blood test in the past 12 months	0.889
Last colonoscopy in the past 12 months	0.494
Last mammogram in the past 12 months	1.000
Last PAP smear in the past 12 months	0.624

A chi^2 test was done in order to compare the DM cohort to the risk cohorts and the p-values are reported.

Supplemetary Table S4. Model Fit Assessment

Variable	p-value
Last influenza shot in the past 12 months	0.811
Intact tetanus immunization	0.851
Intact diphtheria immunization	0.688
Intact Polio immunization	0.902
Intact TBE immunization	0.899
Intact pneumococcus immunization	0.495
Last blood pressure measurement in the past 12 months	0.390
Last blood cholesterol measurement in the past 12 months	0.346
Last blood sugar measurement in the past 12 months	0.602
Last fecal occult blood test in the past 12 months	0.993
Last colonoscopy in the past 12 months	0.247
Last mammogram in the past 12 months	0.859
Last PAP smear in the past 12 months	0.037

Here the Hosmer–Lemeshow test p-values are reported in order to test model fit.
